# The edge effect: A global problem. The trouble with culturing cells in 96-well plates

**DOI:** 10.1016/j.bbrep.2021.100987

**Published:** 2021-03-25

**Authors:** Morva Mansoury, Maya Hamed, Rashid Karmustaji, Fatima Al Hannan, Stephen T. Safrany

**Affiliations:** RCSI-Bahrain, PO Box 15503, Adliya, Bahrain

**Keywords:** 96-Well plates, Cell culture, Edge effect

## Abstract

**Background:**

The use of 96-well plates is ubiquitous in preclinical studies. Corner and edge wells have been observed to be more prone to evaporation compared to interior wells.

**Methods:**

Mammalian cells were cultured in 96-well plates over a period of 72 h. VWR and Greiner plates were tested. MTS reagent was added, and metabolic activity was determined after 2 h.

**Results:**

When using VWR plates, cells showed a highly heterogeneous pattern of cell growth. The outer wells showed 35% lower metabolic activity than the central wells. Cells grown in rows two and three also grew sub-optimally (25% and 10% reduction compared to central wells). Greiner plates showed better homogeneity. Cells grown in the outer wells showed 16% lower metabolic activity while cells in rows two and three showed reductions of 7 and 1%, respectively. This edge effect was partially mitigated by storing the plates in loosely sealed wrapping during incubation. Placing a buffer between the wells of the plate further improved homogeneity for the Greiner plates.

**Conclusion:**

Different brands of 96-well plates show different levels of the edge effect. Some clearly are inappropriate for such studies.

**General significance:**

Each laboratory needs to determine their own optimum conditions for culturing cells empirically before continuing to use multiwell plates. Otherwise, large artifacts may arise, affecting the quality of data, with the potential of introducing type I or type II errors.

## Highlights

• The edge effect is frequently observed when using multiwell plates.
• This effect can extend in as far as three rows on a 96-well plate.
• Rewrapping the plate in its original wrapping does not prevent the edge effect.
• Adding liquid between the wells reduces the edge effect.
• Plates from different manufacturers yield different results.

## Graphical abstract

**Figure:**
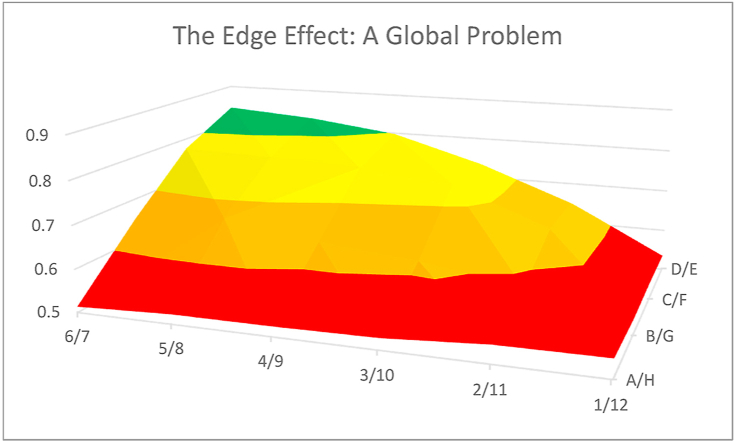

## 1 Introduction

**Fig. 1:**
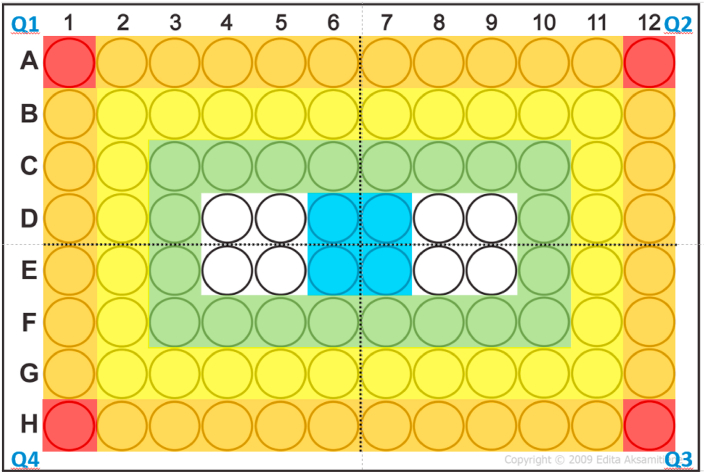
**Designation of well on each plate.** Wells were designated as: Corner (red), Outer Well (orange), 2nd Row (Yellow), 3rd Row (green) or Centre (blue). Template take from http://www.cellsignet.com/media/plates/96.jpg (For interpretation of the references to color in this figure legend, the reader is referred to the Web version of this article.)

The use of multiwell plates is ubiquitous in preclinical studies. They are widely used for assessment in short-term experiments because they allow rapid analysis through multiplate readers. They are also used in long-term experiments in which cells are grown in monolayer on specially treated surfaces. Treatment of cells with antiproliferative agents is the cornerstone of drug discovery in oncology, so it is essential that users understand the limitations of these devices. One notable example is the increased evaporation from corner and edge wells is greater than that from interior wells [1,2] (see Fig. 1 for an explanation of our terminology).

It is widely accepted that the outer wells of multiwell plates offer different conditions to inner wells, although some manufacturers market plates that are claimed to eliminate this problem [3].

Our objective was to study the severity of the edge effect, and whether there was a simple solution to resolve the problem. We aimed to achieve this by looking at the metabolic activity of the cells in the 96-well plates. We measured the metabolic activity of cells using the MTS assay and captured the variation in readings using two brands of 96-well plate. A preliminary description of these data has previously been published in abstract form [4].

## 2 Materials and methods

SW480 colorectal cancer cells (a gift from Dr GB Willars, University of Leicester, UK) were maintained in DMEM high glucose (D5796, Sigma Aldrich) supplemented with 10% foetal calf serum (F7524, Sigma Aldrich) and seeded into two different brands of 96-well plates (VWR, 10062–900 and Greiner, 655180). Each well was seeded with 10,000 cells in 100 μl medium, and plates were incubated for 72 h at 37 ^°^C in a humidified incubator containing 5% CO_2_. To mimic normal incubation conditions in a laboratory, the front door of the incubator was opened 10 times during the incubation period. Plates were either incubated in their original wrapping (Greiner peel-off wrapping, VWR hard plastic) and loosely held together with autoclave tape, or placed directly on incubator shelves. Plates were not stacked. Sterile phosphate-buffered saline (PBS) was also added between the wells of two groups of Greiner plates to determine if this had an effect.

After 72 h, the metabolic activity of the cells was measured with the addition of MTS reagent (10 μl) to each well. MTS reagent was prepared by dissolving 42 mg MTS (Promega G1111) in 2.1 ml DMSO and diluting to 21 ml in PBS. This was mixed with 1 ml PMS solution (prepared by dissolving 0.92 mg PMS (Sigma P9625) in 1 ml PBS). Aliquots were frozen for long-term storage.

Absorbance readings (490 nm) were taken immediately using a Thermo MultiSkan GO Microplate Spectrophotometer. Plates were incubated at 37 ^°^C in a humidified incubator containing 5% CO_2_ for 2 h, and absorbance readings were repeated. Metabolic activity was determined by subtracting time zero reading from the 2-h time point.

Each condition was assessed 6–8 times. Data from each quadrant of each plate were pooled, giving each position four readings per plate (for example, A2, A11, H2 and H11 were comparable). Wells were designated: Corner, Outer Row, 2nd Row, 3rd Row and Centre (Fig. 1). Absorbance values from each of these groups were compared to the mean values of the central four wells (D5:E6) using randomised block one-way ANOVA. Significance of differences was determined using “repeated measures” one-way ANOVA, followed by a Dunnett's *post hoc* test, using GraphPad Prism 7. *P* < 0.05, adjusted to account for multiple comparisons, was considered significant. Our analyses consider each 96-well plate to represent n = 1. This appears not to be the case in some other, earlier publications [1,5], where each well seems to have been considered an individual data point.

## 3 Results

**Fig. 2:**
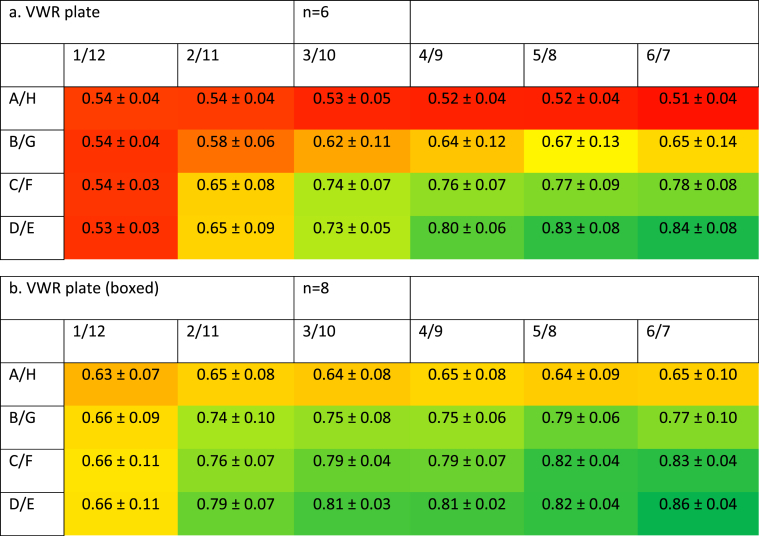
**Profile of metabolic activity of cells grown in VWR plates.** SW480 cells were grown for 72 h before being subjected to an MTS proliferation assay. Data show absorbance (490 nm) increase and standard deviation of sample data following a 2-h incubation in the presence of MTS reagent (calculated using Microsoft Excel) and represent n = 6.2a: incubated alone. 2b: incubated with packaging.

We compared the metabolic activity of SW480 cells 72 h after plating into 96-well plates. Metabolic activity, measured using the MTS assay, showed a large discrepancy between wells. The results were greatly dependent on well location and brand of plates used: in the VWR plates, growth in the corner wells was reduced by 34 ± 2%, mean ± SEM, n = 6 (*P* < 0.0001) when compared to the four central wells. Comparison between the central and the outer wells showed a similar reduction (35 ± 3%, *P* < 0.0001). The second row also showed a significant reduction in metabolic activity (25 ± 5%, *P* < 0.0001) as did the third row with a decrease of 10 ± 5% (*P* = 0.015) (Fig. 2a).

In order to remedy this problem, the plates were inserted back into their original packaging and incubated as above. This was observed to reduce the edge effect: cells in the corner wells showed 26 ± 5% reduced growth (n = 6, *P* < 0.0001). Similarly, the outer (23 ± 4%, *P* < 0.0001) and second (11 ± 5%, *P* = 0.023) rows showed significantly reduced growth. Cells in the third row showed no reduction (5 ± 3%, *P* = 0.36) when compared to the central wells (Fig. 2b).

**Fig. 3:**
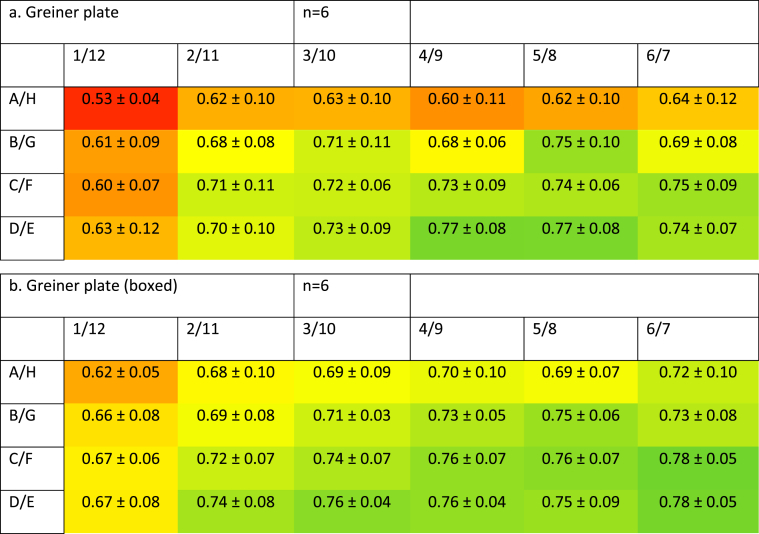
**Profile of metabolic activity of cells grown in Greiner plates.** SW480 cells were grown for 72 h before being subjected to an MTS proliferation assay. Data show absorbance (490 nm) increase and standard deviation of sample data following a 2-h incubation in the presence of MTS reagent and represent n = 6 (3a) or 8 (3b). 3a: incubated alone. 3b: incubated with packaging.

The Greiner brand had more homogenous activity throughout the plate in comparison to the VWR brand. These Greiner plates showed reduced growth in the corner (26 ± 4% reduction, n = 6, *P* < 0.0001) and outer well (16 ± 8% reduction, *P* = 0.0076). Results from the second (7 ± 7% reduction) and third (1 ± 6% reduction) rows showed no decrease (*P* = 0.66 and 1.00, respectively) (Fig. 3a). It is noted that the maximum absorbance was lower in the Greiner plates when compared to VWR plates, suggesting slower growth of cells in these plates.

In order to improve the situation, these plates were also inserted back into their original packaging. Placing the Greiner plates back into their original packaging did not show an improvement. Compared to the central wells, corner wells (19 ± 4% reduction, n = 8, *P* < 0.0001), and outer wells (13 ± 5% reduction, *P* = 0.0004) showed a significant decrease in growth. The second and third rows showed no reduction in growth (5 ± 5% and 2 ± 4%, respectively). These values were not significantly different from growth in the central wells (*P* = 0.070 and 0.84, respectively) (Fig. 3b).

Greiner plates have depressions between the wells that would allow liquid to be placed between the wells. The addition of buffer between the outer wells or all wells was also investigated, to determine if this would minimise heterogeneity.

**Fig. 4:**
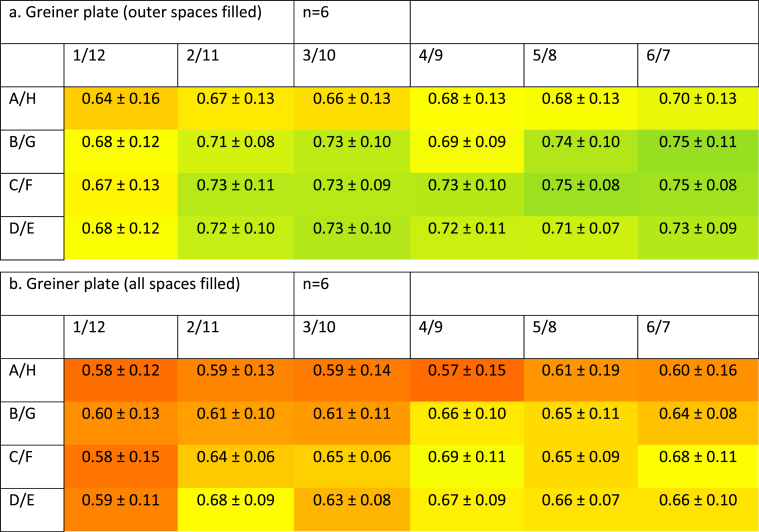
**Profile of metabolic activity of cells grown in Greiner plates with added buffer between the wells.** SW480 cells were grown for 72 h before being subjected to an MTS proliferation assay. Data show absorbance (490 nm) increase and standard deviation of sample data following a 2-h incubation in the presence of MTS reagent and represent n = 6.4a: only outer spaces filled. 4b: all spaces between wells filled.

Filling the outer spaces (around the outer row) of Greiner plates showed improvement. However, growth was still significantly reduced in the corner wells (13 ± 7% reduction, n = 6, *P* = 0.003). Cells grown in the outer wells appeared to show a slight reduction (9 ± 6% reduction, *P* = 0.074), but this was not statistically significant. Cells grown in the second (2 ± 5%) and third (0 ± 4%) rows showed no reduction in growth (Fig. 4a). Further improvements were apparent when all spaces between the wells were filled with buffer. Results from these plates yielded improved performance in the corner and outer wells (13 ± 8% and 10 ± 9% reduction, respectively, n = 6). Statistical analysis showed these reductions were not statistically significant (*P* = 0.083 and 0.18, respectively). Cells grown in the second (2 ± 7%) and third (−1 ± 4%) rows showed no difference from the central wells (*P* = 0.96 and 1.00, respectively) (Fig. 4b). It is notable that cells grown with buffer around each well show lower absorbance, and hence, lower metabolic activity than those without buffer between the wells.

## 4 Discussion

The objective of our study was to measure the change in cellular metabolic activity, rather than evaporation volume, based on well location on a 96-well plate. Differential evaporation of volume from the outer wells to inner wells has been previously reported to affect the result of routine growth inhibition studies of anti-cancer drugs as well as the reading of MTS assay of the outer wells [3,5]. In similar experiments previously performed, others saw a decrease in volume associated with an increase in MTS readout, despite no change in cell number [6], which they explained by an increase in concentration of the MTS reagent. However, as volume decreases, the pathlength of light through the solution would also decrease. Our interpretation of the Beer-Lambert Law (A = εcl, where A = absorbance, ε = molar absorption coefficient, c = concentration of absorbing material, and l = optical pathlength) would anticipate that increases in concentration due to decreases in pathlength would nullify each other. While we cannot explain their results, we recognise they appeared not to have problems with wells on the outer perimeter altering cell growth conditions, as cell counts were equal in all their wells [6]. We appreciate (and show here) different plates have different properties: our Greiner plates outperformed the VWR plates; it is possible that the plates purchased from Asahi Technoglass Corporation (now ATC) [6] outperform both.

In the VWR plates, a significant reduction of metabolic activity towards the outer edges of the plate was observed in comparisons to the central region. This reduction was less in wells closer to the centre. However, a significant decrease was observed in all wells when compared to the central four. Incubating the plates with the original wrapping did reduce the drop in metabolic activity, suggesting evaporation is a factor. However, even with this precaution, a reduction in metabolic activity was statistically significant in the outer two rows*.* This is of great concern, and we do not recommend these plates for use in prolonged (>3 day) studies.

Greiner plates showed a more uniform performance across the wells when compared to the VWR plates overall. The “edge effect” was, however, clearly observed in all outer wells. Incubation of the plates in their original packaging only very slightly improved metabolic activity. The outer wells still showed a statistically significant reduction in metabolism.

The homogeneity was further improved by filling the empty spaces surrounding the well with buffer. Using this approach also decreased the reduction in metabolic activity in the outer wells, with only corner wells showing a statistically significant reduction. However, we would still not rely on data from the outer wells.

It is important to view these results in the context of cancer drug screening, where these discrepancies can skew results and misinterpret the efficacies of drugs. With increased evaporation in outer wells, the test drug concentration would be increased, while the higher concentration of salts in the media would hinder growth simultaneously.

It is common practice to disregard the outer rows or even the outer two rows [6] in similar experiments to minimise the edge effect. This would allow for the use of 62.5% (60/96 wells per plate, excluding outer rows) or 33.3% (32/96 wells per plate, excluding outer two rows) of the purchased plates, increasing cell culture plate costs by approximately 50 and 200%, respectively. Due to a limited sample size (n = 6 or n = 8), some of our data is not “statistically significant,” yet a trend is observed. For this reason, we advise caution.

It is clear that some 96-well plates show a more heterogeneous performance than others. The variation between wells makes some plates totally unsuitable for long-term experiments. Individual end-users will also introduce their own series of other confounding factors. For example, the frequency of opening incubator doors, which causes a loss of humid, CO₂-rich, warm air from the incubator, may reduce the growth rates of the cells. Moreover, different cells may be more or less sensitive to these changes than our SW480 cells. It is recommended that each laboratory observes how its plates respond to their specific environments. This will enable users to minimise the edge effect.

We observed that the MTS readings are higher, overall, in VWR plates compared to the Greiner plates. This suggests that the cells grow more rapidly in the centre wells of VWR plates. It is possible that the rate of heat loss in the VWR plates is less when they are removed from the incubator for handling compared to the Greiner plates, allowing the cells to become more active after MTS is added.

In the Greiner plates, the addition of buffer between the wells reduces the edge effect. However, addition also reduced the MTS signal. We added 9.6 ml of cell suspension originally into plates (96 wells – 12 × 8, 100 μl/well) and 11.7 ml buffer around the well for plates shown in Fig. 4b (117 spaces – 13 × 9, 100 μl/space). As this buffer also cools down while the plate is having MTS reagent added to it, does it require a longer time for the temperature to recover? This could reduce the time the cells are growing optimally in the presence of the MTS reagent. Other considerations may include the time between seeding wells with cells and placing the plates into the incubator. For example, leaving Chinese hamster ovary cells at room temperature for 60 min reduced the edge effect and allowed a more even spread of cells adhering to each well [7]. Similarly, as little as 15 min of incubation at room temperature decreased the edge effect when Vero cells were cultured in CellBIND 96-well plates [8]. This phenomenon appears to be not restricted to only mammalian cells: a 60-min pre-incubation at room temperature greatly reduced the edge effect when *Klebsiella pneumoniae* was cultured in 384-well plates [9].

## 5 Conclusions

In conclusion, in some 96-well plates, the edge effect extends well beyond the edge, making them unsuitable for cellular proliferation assays. Others should be used with caution, and require optimisation before meaningful results can be obtained.

## Author contribution statement

All authors were actively involved in performing the experiments and writing this manuscript.

## Declaration of competing interest

This research was conducted in the absence of any commercial or financial relationships that could be construed as a potential conflict of interest.
